# Acral post-traumatic tumoral calcinosis in pregnancy: a case report

**DOI:** 10.1186/1752-1947-5-89

**Published:** 2011-03-02

**Authors:** Nick Hutt, Davinder PS Baghla, Vivek Gulati, Philip S Pastides, Mike C Beverly, Wassim A Bashir

**Affiliations:** 1 Department of Orthopaedic Surgery, Ealing Hospital NHS Trust, Uxbridge Road, Southall, UB1 3HW, UK

## Abstract

**Introduction:**

Tumoral calcinosis is an uncommon disorder characterized by the development of calcified masses within the peri-articular soft tissues of large joints, but rarely occurs within the hand.

**Case presentation:**

We present the case of a 31-year-old pregnant Indian woman with a three-month history of painful swelling within the tip of her right middle finger following a superficial laceration. She was otherwise well and had normal serum calcium and phosphate levels. Plain radiography demonstrated a dense, lobulated cluster of calcified nodules within the soft tissues of the volar pulp space, consistent with a diagnosis of tumoral calcinosis. This diagnosis was confirmed on the basis of the histopathological examination following surgical excision.

**Conclusion:**

To the best of our knowledge, we present the only reported case of acral tumoral calcinosis within the finger, and the first description of its occurrence during pregnancy. We review the etiology, pathogenesis and treatment of tumoral calcinosis.

## Introduction

Tumoral calcinosis is an uncommon pathological entity characterized by multiple circumscribed, tumor-like, calcified masses in peri-articular connective tissue. These lesions mainly comprise calcium hydroxyapatite crystals and amorphous calcium phosphate [1]. They were originally described by Giard in 1898 [2] and termed *endotheliome calcifie*. The term *tumoral calcinosis *was coined by Inclan *et al*. in 1943 [3], who described a familial condition characterized by normal serum calcium levels and elevated or normal serum phosphate levels. The term "tumoral calcinosis" has also been loosely used to describe secondary metastatic peri-articular calcification occurring in conditions such as renal insufficiency, hyperparathyroidism, hypervitaminosis D and milk-alkali syndrome. These disorders display an underlying abnormality of calcium and phosphorus homeostasis. In contrast, dystrophic tumoral calcinosis occurs as a result of damaged or devitalized soft tissues, but in the presence of normal biochemistry, for example, following trauma, infection, inflammation or neoplasia.

The most frequent cause of tumoral calcinosis is chronic renal failure, with a reported prevalence of 0.5% to 1.2% in patients undergoing hemodialysis [4]. Familial tumoral calcinosis is noted to occur with a significantly higher incidence in patients of African descent but exhibits no sex predominance [5]. The differential diagnosis includes other conditions causing ectopic calcification, such as calcific tendonitis, calcinosis universalis, calcinosis circumscripta, synovial osteochondromatosis, synovial sarcoma, myositis ossificans, tophaceous gout and calcific myonecrosis.

The most common locations of tumoral calcinosis are around the hip joint, elbow, shoulder, foot and wrist joints, with a predisposition for extensor surfaces [1]. This condition does not commonly involve the hand, and to the best of our knowledge, we present the first reported case of a post-traumatic acral fingertip lesion occurring during pregnancy.

## Case presentation

A 31-year-old, right-hand-dominant woman of Indian origin presented with a three-month history of increasing swelling overlying the volar tip of her right middle finger. She recalled sustaining a superficial laceration to the area with a clean kitchen knife, which was treated successfully by application of a clean, dry dressing. She subsequently noticed the gradual appearance and enlargement of a locally painful swelling at her fingertip over a three-month period. Two weeks prior to presentation her general practitioner prescribed antibiotics for a presumed infection, but she experienced no improvement in symptoms. Although she was 30 weeks into her uncomplicated pregnancy at the time of injury, she had no history of systemic disease or any relevant family history.

**Figure 1:**
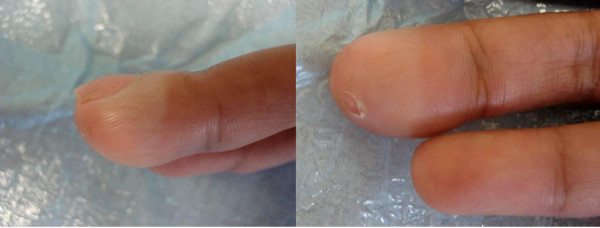
**Pre-operative clinical photographs of finger lesion**.

The clinical examination revealed a tender, solitary 1.5 cm × 3 cm elliptical lesion located within the pulp space of the patient's right middle fingertip and extending to the distal inter-phalangeal joint. The swelling had a firm, non-fluctuant nodular consistency, with an overlying, centrally located punctum from which no discharge was expressible (Figure 1). The patient's hand was neurovascularly intact, with no regional lymphadenopathy present. She maintained a full range of movement at the distal and proximal inter-phalangeal joints.

**Figure 2:**
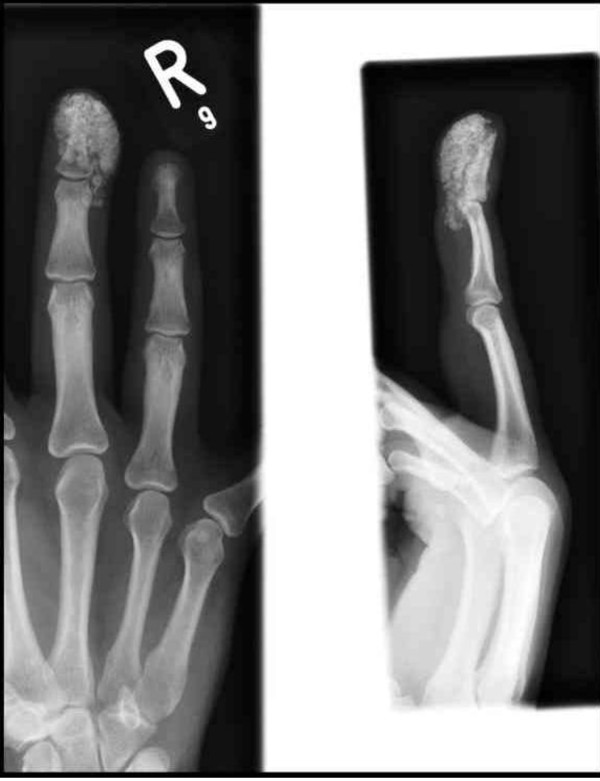
**Pre-operative radiographs**.

The laboratory test results, including complete blood count, urea and electrolytes, erythrocyte sedimentation rate, C-reactive protein, serum urate, calcium, phosphate and alkaline phosphatase were all within the normal ranges. Plain radiographs revealed multiple circumscribed, calcified masses located in the soft tissues adjacent to the distal phalanx involving the distal inter-phalangeal joint with some extension into the distal aspect of the proximal phalangeal region (Figure 2). A diagnosis of tumoral calcinosis was subsequently suspected.

**Figure 3:**
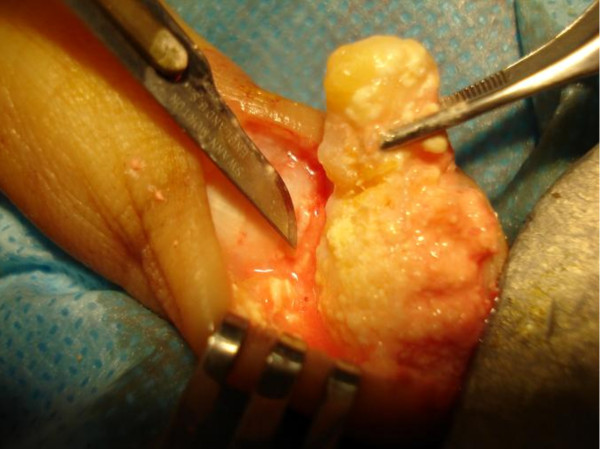
**Intra-operative photograph**.

**Figure 4:**
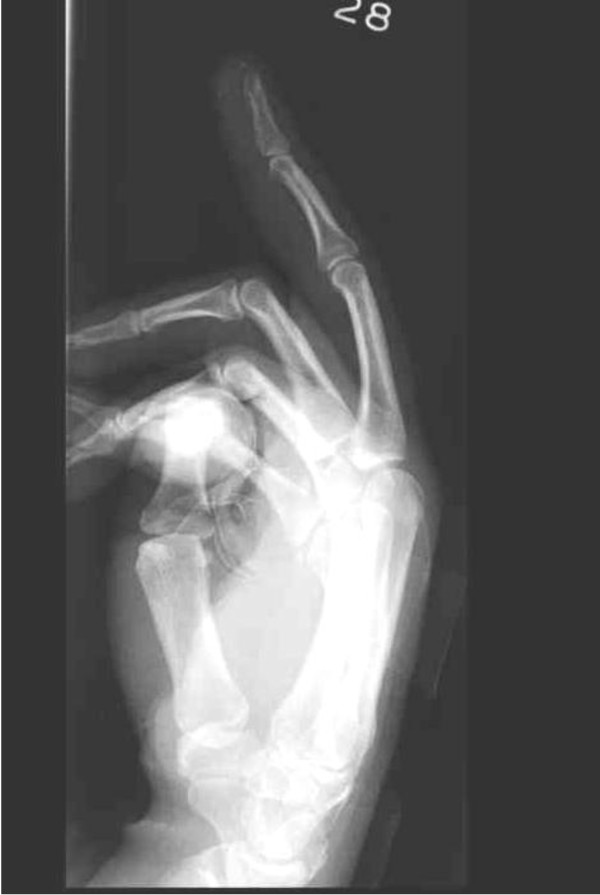
**Post-operative radiographs**.

Surgical excision was performed with the patient under general anesthesia and tourniquet control through a 3 cm volar mid-line longitudinal incision overlying the finger pulp. Macroscopically, three pieces of firm, dense, chalky white nodular tissue ranging from 30 mm × 10 mm × 5 mm to 10 mm × 5 mm × 2 mm were excised and sent for histopathological examination (Figure 3). Microscopically, the specimens consisted of fragmented pieces of dense fibrous tissue surrounding nodular aggregates of heavily calcified debris. Focal foreign body giant cells were seen surrounding the calcification. There was no epithelial element identifiable and no evidence of malignancy. These features were compatible with the radiological diagnosis of tumoral calcinosis. Post-operative radiographs confirmed the successful removal of the calcified tissue (Figure 4), and the patient recovered well with no evidence of recurrence at her six-month follow-up examination.

## Discussion

Since the publication of Inclan *et al*.'s original series [3], more than 300 cases of tumoral calcinosis have been reported. Several cases of hand lesions have been published [6-9], with the lesions most commonly affecting the metacarpophalangeal joints. Although a thumb tip lesion following severe trauma has been described [9], there is no report in the literature of tumoral calcinosis arising *de novo *in the distal phalangeal and/or acral region of a finger alone. Additionally, the case we describe involving the combination of pregnancy with tumoral calcinosis is unique. A distinctive feature of tumoral calcinosis affecting the hand is that it is painful because of the superficial location and pressure on adjacent cutaneous nerves [7].

The literature is inconsistent regarding the diagnostic criteria for tumoral calcinosis, with some texts stating that only the familial form, as originally described by Inclan *et al*. [3], deserves this diagnosis [1]. Smack *et al*. [10], having reviewed the literature, proposed a pathogenesis-based classification system: primary normophosphatemic, characterized by normal serum electrolytes, including calcium and phosphate; primary hyperphosphatemic caused by hypophosphaturia; and secondary tumoral calcinosis.

The etiology of primary as well as secondary tumoral calcinosis remains unknown, although it is accepted that a familial disturbance in phosphate homeostasis is a contributing factor [11]. The mechanism by which tumoral calcinosis is formed appears to be initiated by a localized trigger in the presence of a local or regional deranged metabolic milieu. The chain of events commences with local hemorrhage, which progresses to fat necrosis, collagenization, collagenolysis and ultimately massive calcification. The initial trigger can arise as a result of repetitive microtrauma, peri-articular sheer forces or direct hemorrhage acting as a catalyst in this calcification cascade [12].

The treatment of tumoral calcinosis depends largely on its underlying cause. Surgical excision is the mainstay of treatment, but recurrences are common following incomplete excision or in cases involving actively progressing lesions [5]. Phosphate depletion therapy alone for primary normo- and hyperphosphatemia has demonstrated varying success [13]. Other therapies, including systemic steroid therapy and radiation therapy, are not recommended [14].

## Conclusion

Tumoral calcinosis is an uncommon pathological entity characterized by multiple circumscribed, tumor-like, calcified masses in peri-articular connective tissue comprising calcium hydroxyapatite crystals and amorphous calcium phosphate. It does not commonly affect the hand. To the best of our knowledge, we present the only reported case of acral tumoral calcinosis linked to pregnancy. Surgical excision offers the best treatment, but other therapies have been suggested in the literature.

## Consent

Written informed consent was obtained from the patient for publication of this case report and any accompanying images. A copy of the written consent is available for review by the Editor-in-Chief of this journal.

## Competing interests

The authors declare that they have no competing interests.

## Authors' contributions

NH wrote the case report and treated the patient pre- and post-operatively. DPSB was the treating consultant who performed the operation and was responsible for the patient's care. He also reviewed the article and approved it. VG wrote the article and reviewed the literature. PP wrote the article and reviewed the literature. MCB was the treating consultant who performed the operation and was responsible for the patient's care. He also reviewed the article and approved it. WAB was the consultant radiologist who performed all the imaging and made the radiological diagnosis. All authors read and approved the final manuscript.
